# Examining the use of process evaluations of randomised controlled trials of complex interventions addressing chronic disease in primary health care—a systematic review protocol

**DOI:** 10.1186/s13643-016-0314-5

**Published:** 2016-08-15

**Authors:** Hueiming Liu, Janini Muhunthan, Adina Hayek, Maree Hackett, Tracey-Lea Laba, David Peiris, Stephen Jan

**Affiliations:** The George Institute for Global Health, University of Sydney, Level 10, King George V Building, 83-117 Missenden Rd, PO Box M201, Camperdown, NSW 2050 Australia

**Keywords:** Process evaluations, Primary health care, Complex interventions, Systematic review, Chronic disease, Qualitative

## Abstract

**Background:**

Randomised controlled trials (RCTs) of complex interventions in primary health care (PHC) are needed to provide evidence-based programmes to achieve the Declaration of Alma Ata goal of making PHC equitable, accessible and universal and to effectively address the rising burden from chronic disease. Process evaluations of these RCTs can provide insight into the causal mechanisms of complex interventions, the contextual factors, and inform as to whether an intervention is ineffective due to implementation failure or failure of the intervention itself. To build on this emerging body of work, we aim to consolidate the methodology and methods from process evaluations of complex interventions in PHC and their findings of facilitators and barriers to intervention implementation in this important area of health service delivery.

**Methods:**

Systematic review of process evaluations of randomised controlled trials of complex interventions which address prevalent major chronic diseases in PHC settings. Published process evaluations of RCTs will be identified through database and clinical trial registry searches and contact with authors. Data from each study will be extracted by two reviewers using standardised forms. Data extracted include descriptive items about (1) the RCT, (2) about the process evaluations (such as methods, theories, risk of bias, analysis of process and outcome data, strengths and limitations) and (3) any stated barriers and facilitators to conducting complex interventions. A narrative synthesis of the findings will be presented.

**Discussion:**

Process evaluation findings are valuable in determining whether a complex intervention should be scaled up or modified for other contexts. Publishing this protocol serves to encourage transparency in the reporting of our synthesis of current literature on how process evaluations have been conducted thus far and a deeper understanding of potential challenges and solutions to aid in the implementation of effective interventions in PHC beyond the research setting.

**Systematic review registration:**

PROSPERO CRD42016035572

**Electronic supplementary material:**

The online version of this article (doi:10.1186/s13643-016-0314-5) contains supplementary material, which is available to authorized users.

## Background

### Why is this field of research important?

With a rapidly rising global burden of disease attributed to non-communicable diseases, access to high quality primary health care (PHC) is essential. Complex interventions, defined as ‘*interventions that comprise multiple interacting components, although additional dimensions of complexity include the difficulty of their implementation and the number of organisational levels they target*’, are frequently deployed in an attempt to address health system deficiencies experienced by patients and providers [1]. Choosing a study design to assess effectiveness of complex interventions is not straightforward, and it is recommended to consider randomisation to prevent selection bias and provide robust evidence [2, 3]. Process evaluations, which are typically carried out in conjunction with randomised controlled trials of such interventions, can help explain for whom, how and why a complex intervention had a particular impact [4].

Such evaluations address the question ‘Is this intervention acceptable, effective, affordable and feasible (for me or) for this population?’ [5]. Process evaluations can enable patient-centred care by providing the opportunity for often over-looked patients’ perspectives to be considered. As an example, while a pragmatic trial of a cardiovascular polypill in Australian PHC indicated the polypill was an effective, cost-effective strategy for improving patient adherence and the prescribing of indicated medications, our process evaluation interviews found that clinicians need to consider the polypill strategy alongside other evidence-based strategies. These strategies should cater to specific patient factors such as health literacy, sense of well-being, financial considerations, establishing ongoing respectful clinician and patient relationships and improving accessibility to health care [6].

Despite the generation of good quality evidence, this often does not translate into improved health outcomes [7]. A key barrier in the literature to research translation is cost at different levels, e.g. high outpatient costs for screening to the patient or cost of medications for the programme [8–10]. While health economic evaluations are increasingly being conducted as separate studies to provide evidence of cost-effectiveness to decision makers, there may be cost information that is relevant to the objectives of a process which needs to be investigated. For example, minimising indirect costs to patients is something that is important in understanding why an intervention may be more acceptable to patients compared to standard care. Conversely, for some, indirect costs associated with the intervention may discourage patients from seeking care. These are economic issues which we propose would be important to capture as part of process evaluations but are not strictly captured in health economic evaluations assessing the incremental cost-effectiveness of interventions. It would be pertinent as part of a process evaluation to incorporate relevant cost data from the onset, especially within PHC trials in low- and middle-income countries (LMIC) and in populations which have complex needs and limited funding to be allocated [9]. This would be important as part of a process evaluation, to unpack whether for whom and how an intervention can be implemented into routine practice after the trial is completed. These findings from process evaluations can then inform adoption of interventions into practice and thus the scalability and sustainability of interventions [11].

### What is known about this field currently?

Process evaluation methodology is evolving [4]. Process evaluations were previously synonymous with qualitative research alongside trials and were conducted to provide a deeper understanding of the disease condition, implementation issues and mechanisms of the intervention [12]. However, there is a growing recognition that using qualitative and quantitative data (mixed methods) can help facilitate trial implementation and research translation [13–15]. For instance, stratifying quantitative outcome data by socio-economic status and triangulating it with qualitative interviews, multi-level modelling and embedded cost-analysis in a process evaluation may be useful in determining the relevance and feasibility of a proven effective complex intervention. Using mixed methods, a clearer picture of the intervention may emerge that could aid various stakeholders in their decision-making.

Although ‘one size fits all’ methods or methodologies are not available, various theories or frameworks to enhance implementation research have been used by researchers to assist in their process evaluations. In early 2015, guidance was published by the Medical Research Council (MRC) UK about the planning, conduct and reporting of process evaluations to aid researchers, policy makers and funders [11]. The article described the proposed functions of process evaluations of looking at feasibility and piloting, evaluation of effectiveness and implementation post-evaluation during the different stages of the development, evaluation and implementation of a complex intervention. These functions expanded upon the conventional definition of process evaluations being limited to during trial implementation and defined ‘implementation’ as ‘*the process through which interventions are delivered, and what is delivered in practice*’. For example, during the post-evaluation implementation stage, the authors recommend that the process evaluation serves to explore how there is ‘*routinisation of the intervention into new contexts, and long term implementation/maintenance*’. The authors suggest that this function of the process evaluation is needed as reviews have showed that post-trial, complex interventions are only partially maintained. Key recommendations regarding the planning, design and conduct, analysis and reporting of process evaluations were also discussed in the MRC recommendations [4, 11]. For example, arguments for whether there should be a separation or integration of the process evaluation and outcome evaluation teams were presented. The need to integrate process and outcome data in the analysis and the timing of when process data should be analysed in relation to outcome data were discussed.

The appraisal of the quality of process evaluations has not been straightforward partly because of the variability in methods [11, 16, 17]. Grant et al. in a literature review found that the process evaluations were of poor and inconsistent quality and proposed seven criteria for the reporting of process evaluations including clearly labelling that it is a process evaluation [17]. Other suggestions include appraising the quality of the process evaluation based on the methods used. Given that most process evaluations will have a qualitative component, a set of criteria to examine the quality in the reporting of qualitative research will be relevant to most process evaluations [18, 19].

Dissemination and reporting of findings from process evaluations especially in academic publications can also be difficult due to a variety of reasons including feasibility due to limited resources for research projects, lag time till dissemination of result or publication bias as usually positive outcome trials will be reported but not necessary negative trials [11, 20]. This in turn could limit the likelihood of such relevant findings affecting policy and practice.

### Why do this review?

The George Institute for Global Health has a current programme of research which focuses on addressing NCDs through cost-effective and equitable strategies in primary health care settings including LMIC, and with indigenous populations [21]. Our studies trial complex interventions such as capacity-building initiatives with local providers [22], use of innovative mobile technology [23], and cost-effective generic medications (e.g. polypill) within primary health care settings [24]. We have found that at times, despite acceptability and effectiveness of these strategies, there are significant challenges that impact upon their scale up. These barriers could be cultural, political or institutional factors [25], but an important reason for limited translation seems to lie in the lack of understanding of implementation issues within contextual factors for the different stakeholders (e.g. patient, provider, policy makers). For example, while a trial in India of a clinical decision support system on a mobile tablet improved initial diagnosis and antihypertensive management of trial patients, and was deemed acceptable by end-users, only 35% of patients attended the scheduled 1-month follow-up [23]. Interviews with stakeholders found that limited patient accessibility to medicines and doctors (for a variety of reasons including inadequate staffing, limited primary health care infrastructure) as the key barrier which needs to be overcome. This contrasts with other trials of electronic health tools (e.g. decision support, text messages) in Australia which tend towards generally more positive and sustained results as such presumably because system issues were less of a significant barrier given the universal and subsidised health care available [26–28]. Given the greater burden of early mortality from NCD in LMIC and disadvantaged populations [29], consolidating our findings in this proposed systematic review with an equity-focused lens to better understand how to strengthen PHC within relevant contextual policy and system issues would be useful. Indeed, systematic reviews of interventions in primary health care have concluded that in addition to clinical outcomes, rigorous evaluations of implementation outcomes (e.g. through process evaluations) are needed to ensure changes in practice [30, 31]. We hope that this systematic review will add to the process evaluation methodology and understanding of effective implementation strategies in different PHC settings [32, 33].

### Objectives and key questions

To our knowledge, this is the first systematic review of process evaluations of randomised controlled trial (RCTs) in complex interventions in PHC. For complex interventions, the pre-specification of a theory for how an intervention is expected to work can be highly informative in identifying the mechanisms by which an intervention was hypothesised to have an impact and why it was found to be successful (or not). It provides a framework for assessing the behaviour of individual actors in the implementation of an intervention, potential breakdowns in the interactions between parties and puts into context these actions. Thus, findings from process evaluations from both positive and negative trials can shed light upon implementation facilitators and barriers, which would add to the collective lessons for researchers. Moreover, given that there are numerous theories and frameworks in this area, we thought it would be informative to describe the breadth of methods used and to make some recommendations on evaluation methods that should be incorporated into PEs of complex interventions. Thus, we aim to consolidate the methodology and methods from process evaluations of complex interventions in PHC and their findings of facilitators and barriers to intervention implementation in this important area of health service delivery.

These objectives will be achieved through addressing these questions: (a) Is there and what is the explicit theory behind the conducted process evaluations? (e.g. normalisation process theory, realist framework); (b) What are the methods used in these process evaluations? (e.g. qualitative research through semi-structured interviews, surveys); (c) At what stage is the process evaluation done? (i.e. feasibility and piloting, evaluation of effectiveness, or post-evaluation implementation.); (d) If an aim is stated (i.e. in the evaluation of effectiveness stage), how are the results of the RCT integrated with the findings from the process evaluations?; (e) What are the strengths, limitations and potential solutions identified by the authors in conducting the process evaluations?; and (f) What are the barriers and facilitators to the implementation of complex interventions identified by the authors?

## Methods/design

This systematic review will focus on process evaluations of RCTs of complex interventions addressing chronic disease in PHC. We have described our methods as per Preferred Reporting Items for Systematic Review and Meta-Analysis for protocol (PRISMA-P) recommendations, and this checklist is included as an Additional file 1 [34].

### Eligibility criteria

Definitions as per PICO-D have been adapted for the purpose of this review [20, 35]:

Participants—participants include patients and health providers in the PHC setting addressing the prevalent chronic diseases as defined by the World Health Organisation—cardiovascular disease, chronic kidney disease, chronic respiratory disease, type 2 diabetes mellitus and depression. PHC as defined by the Alma-Ata declaration [36] as health services provided within the community setting by doctors, nurses and allied health with the goal to achieve better health for all through reforms in universal coverage, public policy, service delivery and leadership [37].

‘Intervention’—complex interventions defined as those ‘*interventions that comprise multiple interacting components, although additional dimensions of complexity include the difficulty of their implementation and the number of organisational levels they target*’ within PHC [4]. This includes a single-faceted intervention that requires multiple actors or pathways and thus makes the implementation complex. It is envisaged that the complex interventions for chronic diseases (if not explicitly defined as a complex intervention) will have elements of the Wagner chronic care model such as community support, case management, self-management, facilitated family support, organisational change, delivery system design, decision support for health care providers and clinical information systems [38].

Comparator—not applicable

‘Outcomes’—(1) findings from the process evaluations of stated implementation barriers and facilitators to the complex intervention. (2) The stated strengths and limitations of the process evaluation methodology from the perspectives of the authors. Both findings will be useful for future conduct of complex interventions in PHC in the planning, conduct of process evaluations and in the consideration of intervention implementation and what barriers need to be overcome in different PHC settings.

Timing—years of search from 1998. This was chosen because a systematic review by Davies et al. shows that there was poor use of theory in implementation research until at least 1998 [39].

Design—process evaluations of randomised controlled trials of complex interventions in PHC. Process evaluation as defined by ‘a study which aims to understand the functioning of an intervention, by examining implementation, mechanisms of impact, and contextual factors’ [11]. As discussed by Grant et al., because process evaluations are not clearly labelled as such, qualitative research conducted alongside RCTs with similar aims will be included [17, 40].

Exclusion criteria—articles were excluded if they were not a journal article, not a report based on empirical research (e.g. protocol, editorial), not reported in English and reviews and not human research.

### Search strategy

#### Information sources

Databases reporting academic publications (MEDLINE, SCOPUS, PsychInfo, CINAHL, EMBASE, Global Health.) In order to locate any process evaluations whose findings were not published or missed in the database searches, we will search major clinical trial registries for completed process evaluations (e.g. Cochrane Central Registry of Controlled Trials, EU registry, ANZTRN and clinical trial registry (USA)). Authors will be contacted in regard to the outcomes of the RCT and findings of their completed process evaluations.

A search strategy was developed and adapted for each database with the initial support of a medical research librarian. Search terms were based on the review objectives and early scoping searches (see Additional file 2: search strategy), key words: process evaluations (including programme evaluation, qualitative research), complex intervention (including chronic care model and its components of community support, case management, self-management, facilitated family support, organisational change, delivery system design, decision support for health care providers and clinical information systems), randomised controlled trials, PHC (including family practice, general practitioners) and chronic disease (including cardiovascular disease, chronic kidney disease, chronic respiratory disease, type 2 diabetes mellitus and depression).

### Study records

#### Data management

After the searches, the shortlisted articles will be exported to Endnote. Data will be stored in a common file that is password protected on the Institute’s server that is accessible by the two reviewers. At each stage of the data selection process during the review (e.g. after consolidation of all articles prior to assessing eligibility based on title and abstract), back up files of the endnote database will be made in order to retrace any steps as needed in the review process, and for any third party adjudication.

#### Selection process

Two reviewers will screen all titles and abstracts identifying potential eligible studies based on inclusion and exclusion criteria, and duplicates are to be removed. This will be done independently to reduce the risk of bias. All eligible studies will be retrieved in full text and reviewed by the two reviewers using predesigned eligibility forms (see Additional file 3). Disagreements will be resolved by consensus of a third party in the review team.

#### Data collection process

Data from all included studies will be extracted by two reviewers using the eligibility and data extraction forms. The data extraction forms (see Additional file 4) were partly guided by the MRC recommendation for process evaluations and Grant et al.’s suggested minimal factors for reporting on process evaluations [4, 17]. The forms will be pilot tested by the two reviewers on the same three articles, iterative changes will be made when appropriate and the two reviewers will independently extract data from the rest of the included list of articles.

#### Data items

Variables to be extracted include data on the RCT and its process evaluation: (1) RCT—study design, setting (rural, urban, country), results (positive, negative or equivalent); (2) process evaluation—any published process evaluation protocol or evidence of pre-specified process evaluation in the main trial protocol, or stated aims of the process evaluation (e.g. examining recruitment, or explaining results), the process evaluation theory, justified methods of integrating trial and process outcomes, stage during which the process evaluation is done (feasibility and piloting, evaluation of effectiveness and post-evaluation implementation), methods of analysis and inclusion of costs incurred.

#### Outcomes and prioritisation

The outcomes of interest for our aims are (1) the stated strengths and limitations of the process evaluation methodology from the perspectives of the authors and (2) findings from the process evaluation of stated implementation barriers and facilitators of the complex intervention. Both findings will be useful for future conduct of complex interventions in PHC—in the planning, conduct of process evaluations and when considering the scaling up of an interventions and what barriers need to be overcome in a PHC setting depending on context [1]. For example, the community’s need, the type of model or availability of PHC services will be different in developed settings as compared to LMIC.

#### Risk of bias in individual studies

For this review, we drew on the use of Tong et al.’s criteria for reporting of qualitative studies [19], on Grant et al.’s proposed framework of minimal requirements for the reporting of process evaluations of cluster randomised controlled trials [17] and on MRC recommendations for process evaluations of complex interventions [4]. Combining insights from these papers, a form of appraisal for risk of bias was derived (see Additional file 5). For the purposes of this review in examining the use of process evaluations alongside RCTs in PHC, studies were not excluded based on quality [20]. Instead, the quality of the studies is presented as a risk of bias graph (low, unclear and high risk) [41].

#### Data synthesis

This will involve the aggregation or synthesis of qualitative findings to generate a set of statements that represent that aggregation and categorisation of these findings on the basis of similarity in meaning and contexts. These categories will then be subjected to thematic synthesis in order to produce a single comprehensive set of synthesised findings that can be used as a basis for evidence-based practice. The synthesis of these qualitative data aims to satisfy the criteria established for the reporting of the synthesis of qualitative health research [18]. Abstracted quantitative data (e.g. number of positive trials) will be presented together with a descriptive narrative form including tables and figures to aid in data presentation where appropriate. We will examine how authors address potential bias through a narrative synthesis how well these are reported in the papers and strategies that may have been employed to mitigate this (e.g. triangulation of key findings). Depending on papers included, there may be subgroup analysis of further exploration of any differences of the barriers and facilitators to intervention implementation by context such as indigenous versus non-indigenous and of developed settings as compared to LMIC.

## Discussion

There is a global call for PHC reform in the areas of service delivery, public policy and leadership to enable greater equity and improved health to different populations. To effect this change will require complex interventions involving multiple players (clinicians, community, allied health professionals, policy makers), disciplines (e.g. education, health) and what is successful in one context may not be suitable in another. Process evaluations conducted alongside RCTs of complex health interventions are valuable in determining whether a complex intervention should be scaled up or modified for other contexts.

The conduct of process evaluations is still a dynamic area with no clear defined method, partly due to the spectrum of methods (e.g. observation, interviews and routine monitoring data). De Silva et al. in 2014 outlined the integration of the Theory of Change into the MRC framework for complex interventions, and one of its aims was to combine ‘*process and effectiveness indicators into a single analysis which can help untangle whether, how and why an intervention has an impact in a particular context, and whether it may be suitable for scale up or adaptation for new settings*’ [42]. Moreover, in regard to future scale up of complex interventions, economic issues pertinent to stakeholders (e.g. patients and providers) would be crucial to policy makers and funders—while this has not been traditionally incorporated together with process evaluations, it would be helpful to see if it has been done [35, 43].

Process evaluations of complex interventions have been increasing in recent years and seem to be variable in objectives, methodology and quality. The MRC guidance in the conduct of process evaluations and in the interpretation of the RCT outcomes may be helpful for researchers to aid in the implementation of effective interventions beyond the research setting. This protocol outlines our methods and design in our efforts to systematically consolidate the collective experience of researchers in this field in conducting, analysing and reporting process evaluations by assembling the findings within the MRC’s process evaluation recommendations and to understand previous challenges and potential solutions in the implementation of evidence-based complex interventions in PHC according to context.

## Abbreviations

LMIC, low- and middle-income countries; MRC, Medical Research Council UK; PHC, primary health care; RCT, randomised controlled trial

## Additional files

Additional file 1: PRISMA-P checklist. (DOC 82 kb) Additional file 2: Example of search strategy. (PDF 314 kb) Additional file 3: Eligibility forms. (DOCX 14 kb) Additional file 4: Data extraction form comprising of four tables. (DOC 34 kb) Additional file 5: Form of appraisal for risk of bias. (DOC 33 kb)
