# Supplementary material for: Examining the use of process evaluations of randomised controlled trials of complex interventions addressing chronic disease in primary health care—a systematic review protocol
Source: Syst Rev. 2016 Aug 15;5:138. doi: 10.1186/s13643-016-0314-5 (PMC4986376; doi:10.1186/s13643-016-0314-5)
Supplement: Additional file 2: — Example of search strategy. (PDF 314 kb) [file 13643_2016_314_MOESM2_ESM.pdf]

Wolters Kluwer

Ovid®

My Account JBI Admin Ask a University of Sydney Librarian Support & Training Help Logged in as Ming Liu at The George Institute for Global Health Logoff

Search Journals Books Multimedia My Workspace EBP Tools

Search History (39 searches)*(close)* Remove Duplicates View Saved

	# ▲	Searches	Results	Search Type	Actions
	1	Program Evaluation.mp. or Program Evaluation/	87714	Advanced	Display More »
	2	Program Evaluation/ or process evaluation.mp. or "Outcome and Process Assessment (Health Care)"/	722945	Advanced	Display More »
	3	Qualitative research.mp. or Qualitative Research/	90806	Advanced	Display More »
	4	qualitative.mp.	477736	Advanced	Display More »
	5	Clinical trials.mp. or Clinical Trial/	1848296	Advanced	Display More »
	6	Randomized Controlled Trials as Topic/ or Clinical Trials as Topic/ or randomised controlled trials.mp.	401314	Advanced	Display More »
	7	complex interventions.mp.	2533	Advanced	Display More »
	8	Mental Disorders/ or Primary Health Care/ or Chronic Disease/ or Diabetes Mellitus/ or chronic care model.mp. or Pulmonary Disease, Chronic Obstructive/ or Disease Management/	1449344	Advanced	Display More »
	9	Primary health care.mp. or Primary Health Care/	158339	Advanced	Display More »
	10	cardiovascular diseases.mp. or exp Cardiovascular Diseases/	5530823	Advanced	Display More »
	11	chronic kidney disease.mp. or exp Renal Insufficiency, Chronic/	215546	Advanced	Display More »
	12	exp Pulmonary Disease, Chronic Obstructive/ or Lung Diseases, Obstructive/ or Respiratory Tract Diseases/ or Chronic respiratory disease.mp. or Chronic Disease/	594762	Advanced	Display More »
	13	Obesity/ or Blood Glucose/ or Diabetes Mellitus, Type 2/ or Diabetes Mellitus/ or type 2 diabetes.mp. or Insulin/ or Hypoglycemic Agents/	1698732	Advanced	Display More »
	14	exp Depression/ or depression.mp.	1119824	Advanced	Display More »
	15	general practice.mp. or Family Practice/ or General Practice/	183431	Advanced	Display More »
	16	family physician.mp. or Physicians, Family/	89336	Advanced	Display More »
	17	Social Support/ or Community Health Services/ or Community Mental Health Services/ or community support.mp.	294906	Advanced	Display More »
	18	Social Environment/ or Adolescent/ or Social Support/ or Mental Disorders/ or Family Relations/ or family support.mp. or Family/	3761942	Advanced	Display More »
	19	case management.mp. or Community Mental Health Services/ or "Quality of Health Care"/ or "Delivery of Health Care"/ or Mental Disorders/ or Case Management/ or Chronic Disease/ or Patient Care Team/	1303857	Advanced	Display Delete More »
	20	self management.mp. or Self Care/	89895	Advanced	Display More »
	21	Primary Health Care/ or "Delivery of Health Care"/ or Organizational Innovation/ or organisational change.mp.	426297	Advanced	Display More »
	22	Primary Health Care/ or Quality Assurance, Health Care/ or Chronic Disease/ or delivery system design.mp. or Diabetes Mellitus/ or "Delivery of Health Care"/	1432354	Advanced	Display More »
	23	Decision Making/ or Decision Support Techniques/ or Decision Making, Computer-Assisted/ or Decision Support Systems, Clinical/ or decision support.mp.	347732	Advanced	Display More »
	24	Information Systems/ or Medical Records Systems, Computerized/ or clinical information systems.mp. or Electronic Health Records/	120031	Advanced	Display More »
	25	1 or 2 or 3 or 4	1206468	Advanced	Display

CONTRACT

					More ≫
☐	26	5 or 6 ▶	1976737	Advanced	Display More ≫
☐	27	7 or 17 or 18 or 19 or 20 or 21 or 22 or 23 or 24 ▶	5923838	Advanced	Display More ≫
☐	28	Primary Health Care/ or Chronic Disease/ or Diabetes Mellitus/ or chronic care model.mp. or Pulmonary Disease, Chronic Obstructive/ or Disease Management/ or Models, Organizational/ ▶	1240820	Advanced	Display More ≫
☐	29	27 or 28 ▶	6042204	Advanced	Display More ≫
☐	30	8 or 10 or 11 or 12 or 13 or 14 ▶	8720590	Advanced	Display More ≫
☐	31	9 or 15 or 16 ▶	391662	Advanced	Display More ≫
☐	32	25 and 26 and 31 ▶	2174	Advanced	Display More ≫
☐	33	29 or 30 ▶	12696033	Advanced	Display More ≫
☐	34	32 and 33 ▶	1588	Advanced	Display More ≫
☐	35	limit 34 to english language ▶	1515	Advanced	Display More ≫
☐	36	limit 35 to ("review articles" and "topic reviews (cochrane)") [Limit not valid in CCTR; records were retained , Limit not valid in Embase,PsycINFO,Global Health; records were eliminated] ▶	8	Advanced	Display More ≫
☐	37	35 not 36 ▶	1507	Advanced	Display More ≫
☐	38	limit 37 to humans [Limit not valid in CCTR,PsycINFO,Global Health; records were retained] ▶	1498	Advanced	Display More ≫
☐	**39**	**remove duplicates from 38** ▶	**1362**	**Advanced**	Display More ≫

Remove Selected | Save Selected | Combine selections with: And Or RSS

Save Search History

Advanced Search | Basic Search | Find Citation | Search Tools | Search Fields | Multi-Field Search

5 Resources selected | Hide | Change

EBM Reviews - Cochrane Central Register of Controlled Trials January 2016, **Embase** 1974 to 2016 February 18, **Ovid MEDLINE(R)** 1946 to February Week 2 2016, **PsycINFO** 1806 to February Week 2 2016, **Global Health** 1973 to 2016 Week 05

Enter keyword or phrase (* or $ for truncation) ◉ **Keyword** ○ Author ○ Title ○ Journal

Search

Limits *(close)* ☐ Include Multimedia

☐ Abstracts ☐ All Journals ☐ English Language
☐ Human ☐ Latest Update ☐ Peer Reviewed Journal

Publication Year - -

Additional Limits | Edit Limits

To search Open Access content on Ovid, go to Basic Search.

Results Tools

Options

☐ **All** | *Range* Print Email Export Add to My Projects Keep Selected

Clear **View:** Title | Citation | Abstract 25 Per Page 1 Go **Next >**

Search Information

You searched:

remove duplicates from 38

- *Search terms used:*

2
(health
adolescent
agents
and
as
assessment
assurance,
blood

1. ☐ Pre-randomization decisions and group stratification in a randomized controlled trial to improve prescribing.

+ My Projects + **Annotate**

2. ☐ Effectiveness of point-of-care testing for therapeutic control of chronic conditions: results from the PoCT in General Practice Trial.

+ My Projects + **Annotate**

3. ☐ Psychiatric consultation in somatization disorder. A randomized controlled study.

+ My Projects + **Annotate**
