# Supplementary material for: Examining the use of process evaluations of randomised controlled trials of complex interventions addressing chronic disease in primary health care—a systematic review protocol
Source: Syst Rev. 2016 Aug 15;5:138. doi: 10.1186/s13643-016-0314-5 (PMC4986376; doi:10.1186/s13643-016-0314-5)
Supplement: Additional file 3: — Eligibility forms. (DOCX 14 kb) [file 13643_2016_314_MOESM3_ESM.docx]

**Additional file 3: Eligibility Criteria Forms**

| **Inclusion Criteria** | **Study** |
| --- | --- |
| Type of study:  Process evaluation  Qualitative study within RCT |  |
| Design:  RCT  Complex intervention |  |
| Setting: primary health care |  |
| **Exclusion criteria** |  |
| Not a journal article, not a specific trial, not a report based on empirical research (e.g. protocol, editorial), not reported in English, and not human research.  Other |  |
