# Supplementary material for: Examining the use of process evaluations of randomised controlled trials of complex interventions addressing chronic disease in primary health care—a systematic review protocol
Source: Syst Rev. 2016 Aug 15;5:138. doi: 10.1186/s13643-016-0314-5 (PMC4986376; doi:10.1186/s13643-016-0314-5)
Supplement: Additional file 4: — Data extraction form comprising of four tables. (DOC 34 kb) [file 13643_2016_314_MOESM4_ESM.doc]

**Additional file 4: Data extraction tables**

**Table 1: Details of the RCTs and its complex intervention**

| **Article** | **Summary of trial being evaluated** | **Causal assumptions clarified (hypothesis of how the intervention would work) (Y/N)** | **Setting (Rural, Urban, Countries** | **Disease (eg mental health, diabetes)**  Mental health | **Main trial outcomes positive/ negative/equivalent** | **Cost analysis (Y/N/NA)** |
| --- | --- | --- | --- | --- | --- | --- |

Table 2: Details about the process evaluation

| **Article** | **Labelled as a process evaluation (Y/N)** | **Stated purpose (Y/N)** | **Pre-specified protocol (Y/N)** | **Processes examined at which stage**  **a) Feasibility and piloting- Acceptability, Testing of processes, Feasibility**  **b) Evaluation of effectiveness- are the main trial designs and findings reported (Y/N/NA), Fidelity, mechanism, contextual influences**  **c)Post-evaluation implementation- integrating of intervention into new context, long term maintenance** | **Specified theory (Y/N)**  **(Theory e.g. Realist)** | **Methods used (eg stakeholder interviews, routine monitoring data, documentary analysis, observations)**  . | **Analysis**  **(if applicable -quantitative data on fidelity dose, reach**  **-detailed modelling across sites**  **-integration of quantitative process data and outcome datasets**  **-qualitative and quantitative analysis building on each other**  **-analysing process data prior to trial outcomes**  **-generating hypothesis or post hoc explanation)** |
| --- | --- | --- | --- | --- | --- | --- | --- |

Table 3: to learn what the strengths and limitations of the PE in these contexts and to discuss what can be used to overcome it eg. Sampling, resources

| **Study** | **Strengths of process evaluation** | **Limitations of the process evaluation** |
| --- | --- | --- |
| For example: Boase 2011 | Insights provided by the SAMS researchers of the trial may have enhanced data quality. | Interviews conducted by the researchers and thus known by the practice nurses, and different data may have been collected otherwise.  Conducted at the end of the trial, and if interviewed at the start may have provided data to address the findings of competing time and demands during the implementation of the trial. |

Table 4: to provide information so as to learn from previous process evaluations in implementing future trials in these contexts

| **Study** | | | **Implementation Issues (stated themes)** | | | | | | **Implementation barriers** | | | **Implementation facilitators** |
| --- | --- | --- | --- | --- | --- | --- | --- | --- | --- | --- | --- | --- |
| For example: Boase 2011 | | | Organisation of research  Delivering the intervention | | | | | | Recruitment into the study and whether it included the practice nurse’s opinion at the start.  Time issues- not having it compensated adequately to deliver the intervention and to do the administrative tasks associated with the study. This at times led to resentment from other team members who perceived that clinical time was taken up by research time resulting in increased work pressure overall.  Competing demands- from GP, patients, research  Standardised script to have it the same across the sites meant that at times it was not patient centred, and at times made the interactions with established patients awkward. | | | Having the buy in of the practice nurse from the start of the study  The intervention and the allocated time provided an opportunity to do things differently, and to allow for patient empowerment and patient centred care. Thus for most of them, positively changed their practice.  Having time set aside for the practice nurse was perceived positively. |
|  |  |  | |  |  |  |  |  | |  |  | |
