# Supplementary material for: Examining the use of process evaluations of randomised controlled trials of complex interventions addressing chronic disease in primary health care—a systematic review protocol
Source: Syst Rev. 2016 Aug 15;5:138. doi: 10.1186/s13643-016-0314-5 (PMC4986376; doi:10.1186/s13643-016-0314-5)
Supplement: Additional file 5: — Form of appraisal for risk of bias. (DOC 33 kb) [file 13643_2016_314_MOESM5_ESM.doc]

**Additional file 5**: Appraisal form for the Risk of Bias

| **Criteria** | **Study** |
| --- | --- |
| **Planning:** |  |
| Degree of separation between outcome and process evaluation teams stated and described. |  |
| **Design and conduct:** |  |
| Process evaluations should clearly state their purpose. |  |
| The intervention should be clearly described and causal assumptions clarified |  |
| Process evaluations should state the choice of methods and justify them in terms of the stated aims of the evaluation, and the selected timing (eg retrospective data collection, was it planned initially) |  |
| If the process evaluation is done at the evaluation stage:  Transparently report of the process data are analysed blind to trial outcomes or for post- hoc explanation |  |
| If qualitative methods used, the study was appraised with the use of COREQ (Domain 1: research team and reflexivity, Domain 2: study design, Domain 3: analysis and reporting) |  |
| **Reporting:** |  |
| Process evaluations should be clearly labeled |  |
| Publish a full report of evaluation components or a protocol paper |  |
| **Risk of Bias**  Low, Unclear, High |  |
